# Supplementary material for: Rapid carbon accumulation at a saltmarsh restored by managed realignment exceeded carbon emitted in direct site construction
Source: PLoS One. 2022 Nov 30;17(11):e0259033. doi: 10.1371/journal.pone.0259033 (PMC9710768; doi:10.1371/journal.pone.0259033)
Supplement: S1 File — (PDF) [file pone.0259033.s001.pdf]

SUPPLEMENTARY INFORMATION

Table S1. Field sampling dates and information. Samples highlighted in bold are those selected for the quantification of total organic carbon. Access issues prevented sampling at some locations in March 2015 and September 2016. We assessed the consequence of the additional uneven sampling by removing samples from these two time periods and recalculating the mean carbon content in newly accreted sediment. The value differed by less than 1% of the original value (i.e. 4.367% vs 4.372%), so we retain all samples in the data presented in the manuscript.

Date of sampling	Days after restoration	Cores sampled	Notes
28 August 2014	-7	Site A1, A2<br>Site B1, B2<br>Site C1, C2, C3<br>Site D1, D2, D3	Pre-restoration sampling. Natural marsh not sampled.<br>A3 not sampled as it was undergoing earthworks.
14-15 December 2014	+101	Site A1, A2, A3<br>Site B1, B2<br>Site C1, C2, C3<br>Site D1, D2, D3<br>NAT	Installation of sedimentation pins
16-17 March 2015	+193	Site A2<br>Site C1, C2, C3<br>Site D1, D2, D3<br>NAT	Access issues prevented sampling at Site B, A1 and A3.
25-28 June 2015	+294	Site A1, A2, A3<br>Site B1, B2<br>Site C1, C2, C3<br>Site D1, D2, D3<br>NAT	
14-15 April 2016	+588	Site A1, A2, A3<br>Site B1, B2<br>Site C1, C2, C3<br>Site D1, D2, D3<br>**NAT**	
27-28 September 2016	+754	Site B1, B2<br>Site C1, C2<br>Site D1, D2, D3	Access issues prevented sampling at Site A and C3.
5-7 March 2017	+913	Site A1, A2, **A3**<br>Site B1, **B2**<br>Site C1, C2, **C3**<br>Site D1, **D2**, D3	Sedimentation pin data collected

Table S2. Summary of the fuel (diesel) consumption and tCO_2e emitted by machinery in the construction of Steart Marshes earthworks.

**Equipment/Component**	**Average fuel burn ($l.hr^{-1}$)**	**Productivity**	**Fuel use (l)**	**t CO_2**	**tC**
Excavator (EC250DL)	20.8[2]	$80 m^3/hr$[6]	127,255	341.0	93.0
A25D ADT	30[1]	10 km/hr[5]	208,689	559.3	152.5
D6 Bulldozer	19[3]	$45.7 m^3/hr$[7]	203,488	545.3	148.7
Roller	1.9[4]		11,580	31.0	8.5
Managed Realignment Earthworks			551,012	1,476.7	402.7
Other Earthworks			110,202	295.3	80.5
**Total Earthworks**			**661,214**	**1,772**	**483**

[1]https://www.equipmentworld.com/owning-and-operating-costs-5;[2]https://www.volvoce.com/en-us/government/services/promotions/fuel-efficiency-guarantee; [3]https://www.constructionequipmentguide.com/industry-begins-grappling-with-rising-fuel-costs/7008; [4] https://www.equipmentworld.com/owning-and-operating-costs-6/; [5] Estimated from average speed on site (obtained from the contractor); [6] Hydraulic Excavator with a 0.5 m^3 bucket excavating clay/chalk, https://www.methvin.org/construction-production-rates/excavation/bulk-excavation; [7] 1.2 m^3 bucket, reach of 50-200 m^2, Boulder Clay https://www.methvin.org/construction-production-rates/excavation/bulk-excavation

Distance travelled

411,397m^3 of material were transported to create the sea defences of the site. The Volvo A25D Articulated Dumper Trucks that were used to transport the material from the dug creek network to the sea walls could carry 10.7 m^3 of sediment (15 m^3 standard volume but including a bulking factor of 1.4 for a clay material, where bulking factor = volume after excavation/volume before). The distances from excavation sites (creek networks) to the dumping sites (points along the new sea defence) were modelled prior to site construction to ensure that there was sufficient material, and these distances were used to quantify the total distance travelled. The bulk of the unsuitable material from the creek excavation was towards the northern end of the scheme. The design of the Steart scheme sought to minimise distances travelled and therefore accommodated more of the landscape fill in the northern end of scheme.

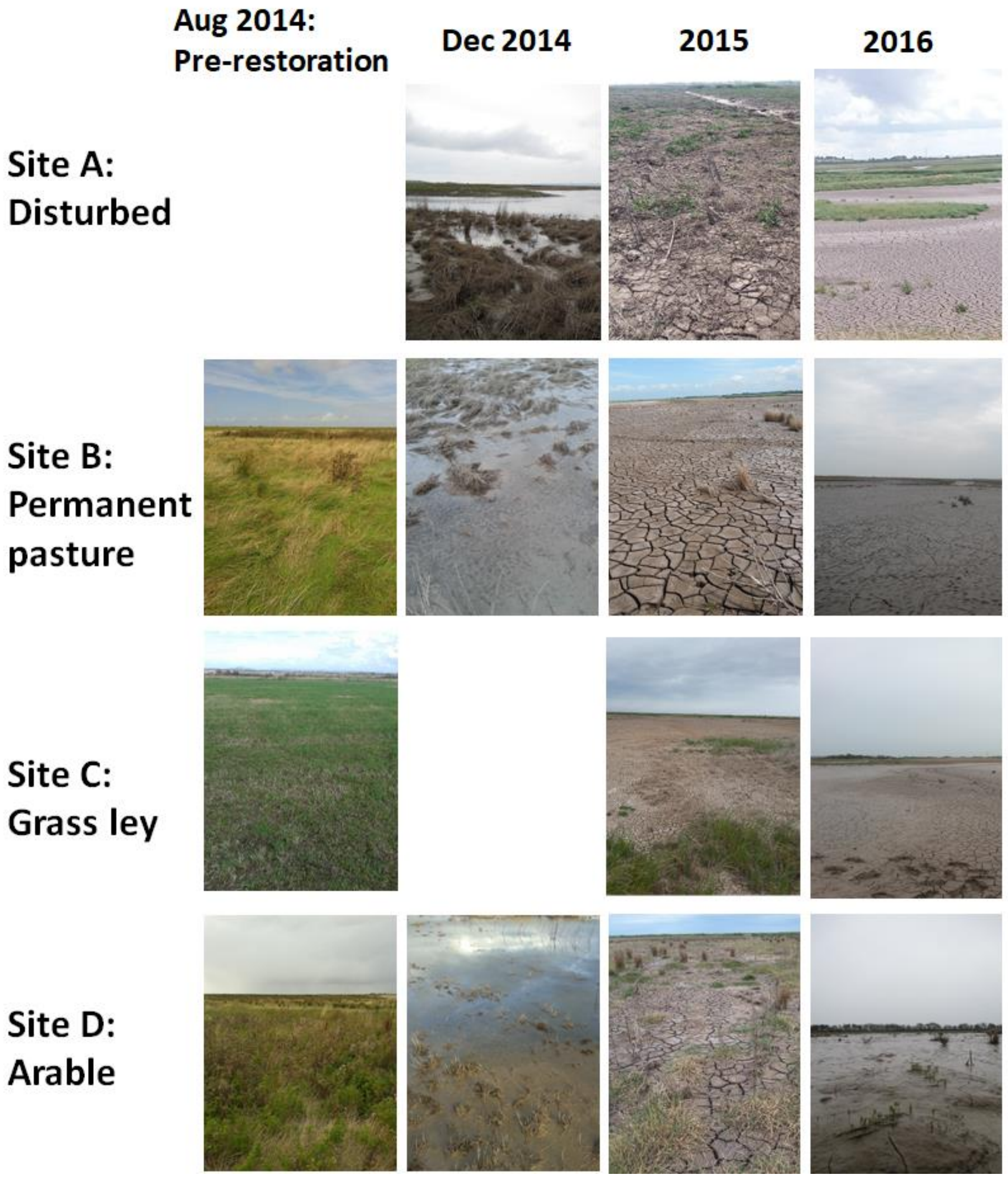

Figure S1. Photographs of sampling areas (Sites A-D). Note sediment cracking in 2015 images when conditions were dry in June; cracks and the 'polygons' formed by the cracking were sampled (see methods)

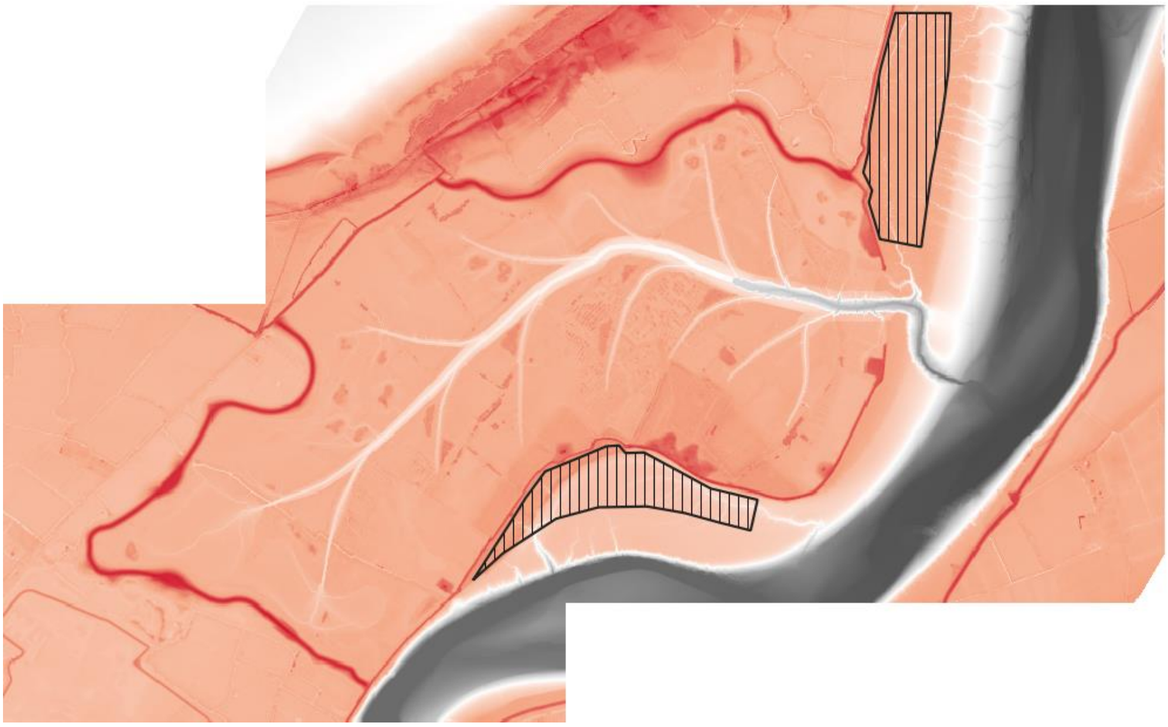

Figure S2. Location of natural marsh polygons used to quantify elevation change of natural marsh. Plotted onto a base map of the 2018 Lidar imagery.

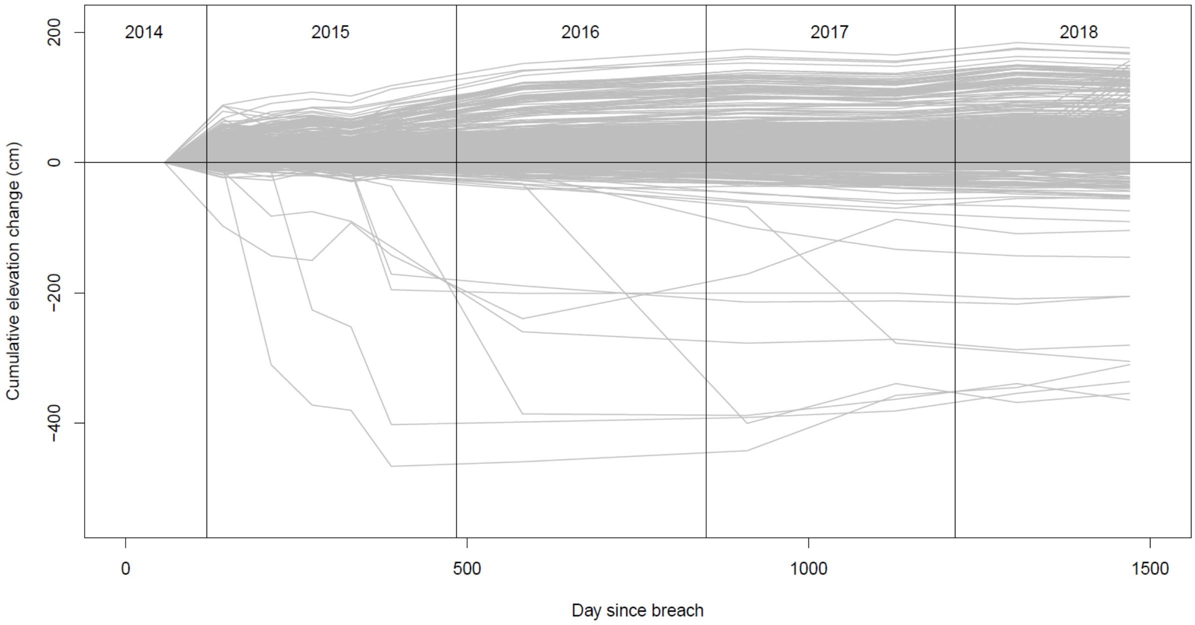

Figure S3. Cumulative elevation change trajectories of a sample of 1000 DTM pixels.

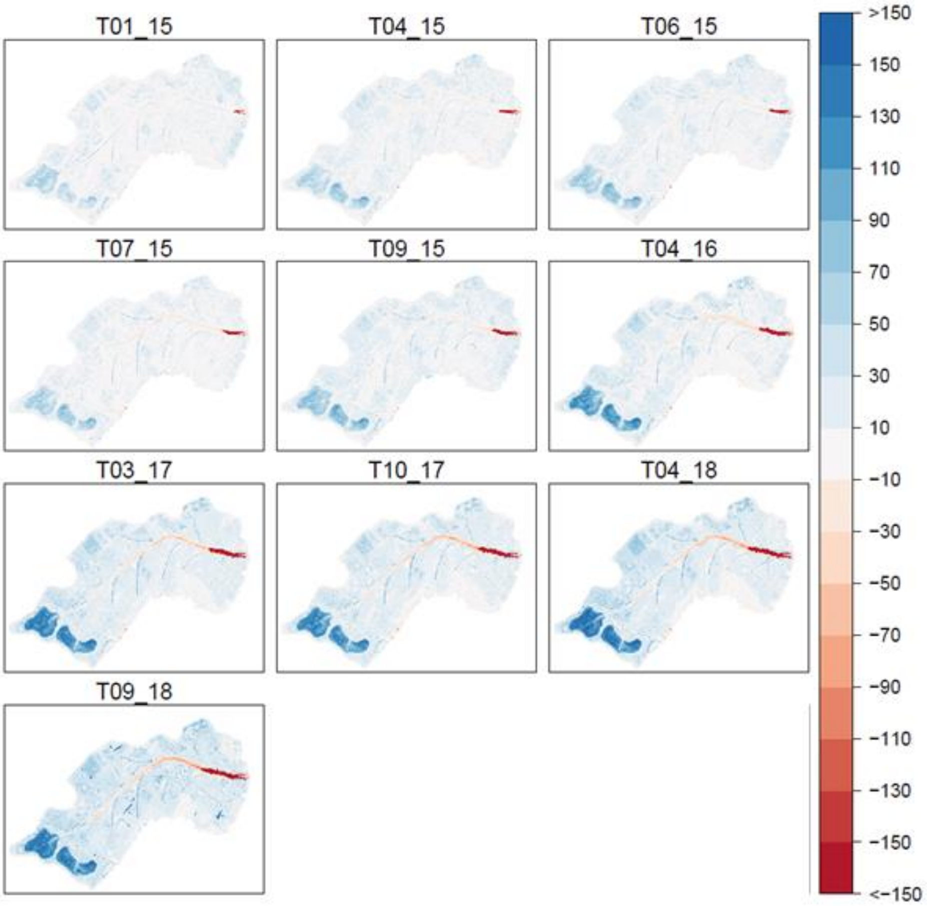

Figure S4. Cumulative change in elevation for each LiDAR survey.
