# Supplementary material for: Rapid carbon accumulation at a saltmarsh restored by managed realignment exceeded carbon emitted in direct site construction
Source: PLoS One. 2022 Nov 30;17(11):e0259033. doi: 10.1371/journal.pone.0259033 (PMC9710768; doi:10.1371/journal.pone.0259033)
Supplement: Attachment — Submitted filename: Response to reviewers.docx [file pone.0259033.s003.docx]

**Response to reviewers**

*We thank both reviewers for their constructive comments on our manuscript, and apologise for the time delay in returning this to you (after your rapidity) due to serious illness of the corresponding author. In this revised manuscript, we have added new analyses suggested by the reviewers, moved the analysis of elevation change into the main manuscript, and expanded the text to address issues raised by the reviewers. Our point by point response to each comment are below.*

Editor comments

The two reviewers I invited requested major revisions for the manuscript and I concur with them. Both referees agreed that the research itself is timely and worthwhile, but they still had a number of critical concerns that I would like you to elaborate in your revision. Among the concerns, I especially note the following items:

- more spatially explicit analysis of the data

*RESPONSE: We have added new analyses to further explore spatial variation in our data. Please see our response to R1’s first comment.*

- data comparability issue

*RESPONSE: TC and TOC were both based on newly accreted sediment so are comparable. Please see our response to R1’s third comment.*

- title and abstract are misleading

*RESPONSE: We agree with R2’s concerns that there are some unresolved uncertainties that may affect the statement that carbon accumulation far exceeds construction costs. We have therefore changed the title to be clear that our results relate to the direct costs of construction. We have also added a caveat about these uncertainties to the abstract and the conclusion.*

- quality of your elevation-change assessment

*RESPONSE: Following R2’s suggestion we have moved our evaluation of this into the main results section. The elevation changes at this site using Lidar has also been validated against fixed control points in a previous study (Serato & Pontee, 2019).*

Reviewer #1:

This manuscript provides data on the topical activity to breach levee banks to achieve carbon sequestration through re-establishing tidal wetlands. Particular consideration is given to how carbon sequestered compares to the carbon emissions arising from the engineering and monitoring work for the managed realignment, as such quantifications of greenhouse gas emissions are needed for carbon market mechanisms. The manuscript is a worthy and timely contribution which will add to the growing body of knowledge on Blue Carbon. The study includes data from a site with several years of tidal inundation, and a comprehensive data set from airborne bathymetry to ground surveys of soil carbon. The manuscript is well written and presented, and relevant literature considered. I recommend publication, after some revision addressing some concerns and missed opportunities on data analyses as explained below.

1). The field sampling includes several sites within the study area (Line 126), separated further by elevation differences. Yet in most of the analyses, these sites are combined and an opportunity missed to present differences arising in the development of soil organic carbon stocks subject to the prior land use as well as elevation, which would affect colonising saltmarsh, inundation and sedimentation. This seems an opportunity lost, and I like to encourage the authors to present more spatially explicit findings of their data.

*RESPONSE: In the original manuscript we assessed how TC proportion in new sediment varies between sampling locations. We have added new analyses of (1) how the ratio TOC:TC ratio varies between sampling locations, (2) how sediment bulk density varies between sampling locations, (3) how TC varies with elevation and (4) how TC varies over time (insufficient data to do 3 and 4 for BD and TOC). We have also added new analysis of how sedimentation rates vary with starting elevation to complement the existing analysis of changes over time. These analyses show:*

- *Sedimentation rates were slower at higher elevations*
- *Sediment carbon content did not significantly change with elevation (but tended to be lower at higher elevations)*
- *The arable prior land-use had the highest TC, and the permanent pasture prior land-use had the highest ratio of TOC to TC*
- *Bulk density did not differ between prior land-uses*
- *Sediment carbon did not change with time or with depth*

*We are cautious about attributing a causal link between prior land-use and carbon content of new sediment as effects were weak, not consistent between TC and TOC, and the depth of new sediment means that it is unclear how past land-use could affect the new sediment.*

2a). Due to the developments at the site following tidal inundation, soil sampling methods varied (Lines 141 onward), and it should be explained or discussed how this affected the assessment.

*RESPONSE: Most post-breach samples were collected by coring. In a limited number of cases, coring resulted in compaction (wet sediments) or sample loss (dry friable sediments). In these cases, we followed best practice as set out in the Coastal Blue Carbon methods manual to avoid/minimise and correct for compression. For cases where compression was minimal and clearly occurred in the surface 10cm (e.g. the core compression was visible when the auger barrel had been inserted to 10cm depth with no further compression as the core is inserted deeper into the profile), we retained the core, applied a compaction correction factor to the surface 10cm (see Coastal Blue Carbon handbook), and also collected an uncompressed surface profile (~10cm) using an adapted syringe (as used for bulk density sampling). When the surface sediments were highly cracked and taking a core resulted in significant compression or sample loss, the simplest way to ensure collection of the surface sediments was to lift out and section an entire sediment plate, and then take a core from the base of the mud-cracks (correcting core depth to depth from surface), where this process avoided any compression or sample loss. As such, the only effect on the assessment is to avoid errors due to sample loss or core compression (i.e. incorrect recording of sample depth or the collection of a bulk density sample from a compressed core). We have made changes to the text to explain this more fully (L143-158)*

2b) For the soil carbon measurements and calculations, international methods (e.g. the Blue Carbon manual) should be applied, which provide established guidance for methods. The calculations for site level carbon (Line 240) appear a bit unusual compared to methods in other Blue Carbon projects.

*RESPONSE: The Blue Carbon Manual suggests a range of methods for measuring soil carbon which include the approach we use (decarbonation, please see our response to R2’s L177 comment for justification of this approach). We apologise that we did not originally cite the Blue Carbon Manual, which was an oversight as it informed our methods.*

***Soil carbon measurements:*** *The Coastal Blue Carbon manual identifies elemental analysis as a technique that provides a quantitative measure of carbon content and states that it is the most suitable method for routine analysis of total carbon p.57-58). In comparison, LOI and the Walkley-Black method are identified as only semi-quantitative measures of organic carbon content. LOI is only recommended if there is no access to elemental analysis / the costs of elemental analysis are prohibitive.*

*The handbook identifies two methods to correct total carbon measured by elemental analysis for soil carbonate content – the first is acidification, where the inorganic carbon content is estimated from the difference in weight of the subsample before and after treatment. The second is to ash the sample, and measure the inorganic carbon in the ash using elemental analysis. In both these cases, the organic carbon content is estimated by subtracting the inorganic carbon content from the total carbon content.*

*Our method is a combination of these two approaches, where we acidify the sample AND then reanalyse the sample using elemental analysis. I.e. we measure the organic carbon directly, rather than calculating it from a measure of the inorganic carbon content. For our own laboratory standards, we have compared the organic carbon contents obtained from our method to those estimated from the acidification mass loss (handbook approach 1), where the organic carbon contents determined from re-analysis are consistently higher than those calculated from mass loss. We attribute this to the loss of other labile phases (e.g. Fe-Mn oxyhydroxides) during acidification. Thus attributing all mass loss to carbonates results in an overestimate of inorganic carbon, and an underestimate of organic carbon.*

***Calculations for site level carbon:*** *In terms of scaling up to the site level, some modification of the Coastal Blue Carbon Manual method is needed as we are interested in quantifying the carbon that has been accumulated in the newly deposited sediments since restoration, not how much carbon the site stores to a stated depth. In effect rather than measuring carbon down to the parent material, we measure carbon down to the agricultural horizon, i.e. the full depth of the newly deposited sediment. We quantify the depth of sediment across the whole site using Lidar. Although this approach is not (currently) included in the Coastal Blue Carbon handbook, the value of remotely sensed data is identified, and it is acknowledged that airborne Lidar may be used to estimate ground elevation accurately (p. 164, and it is regularly used for this purpose elsewhere).*

*The Coastal Blue Carbon manual recommends a 3-dimensional assessment of carbon stocks – by site stratum and depth interval. Our treatment of the new sediment as a single population is justified by the minimal differences observed in carbon content between sampling locations (for which the explanation is currently uncertain, and therefore does not provide a robust basis on which to stratify the site) and the uniformity of carbon content with depth in the sediment. We note that as vegetation becomes more established across the site, clearer differences in organic carbon content may arise and it will likely become appropriate to stratify the site by vegetation community and depth. A benefit of our approach is that it also allows error propagation of both uncertainty in sediment carbon density and sediment volume, whereas the Blue Carbon Manual approach just quantifies uncertainty in sediment carbon density.*

3). Clarification is needed on the different approaches used for total carbon (TC) and total organic carbon (TOC). TC data are calculated for all depths sections to 50 cm (Line 157), whereas TOC data are calculated for the depths of the sediment horizon which has accreted over time since tidal inundation (Line 171). To set the soil carbon developments in context with managed realignment, taking the sediment be the horizon of the previous land use is fine (Line 229), but it has to be done for both TC and TOC, otherwise the data are not comparable.

*RESPONSE: TC and TOC are both quantified for newly accreted sediment above the horizon, so are directly comparable. Furthermore, the TOC/ TC ratio has been calculated on a per sample basis so relates to TOC and TC measurements from the same sediment samples. We did take additional TC measurements from below the horizon, but these are not used to calculate TC content of new sediment (see existing text L242-245).*

4). In the course of colonisation and land use change following the opening of the levee bank, a dynamic change of sedimentation/erosion and recolonisation would have occurred and resulted in a differentiated pattern of carbon sequestration. The authors are not exploring this at all, but instead lump all data together across locations, times and depths (Line 227) and also across years (Line 235). The reasoning for considering this amalgamation of data since tidal inundation needs to be added, why was it justified (Line234)? Table 1 includes data from the single sampling times since the breach occurred, and more can be done with the data on increase in TOC over time, and resulting changes in the TOC/TC ratio in the course of recolonisation with saltmarsh.

*RESPONSE: We assessed whether sediment carbon content varied over time (L362-363), with depth (Fig. 6, L246), with prior land-use (L355-357) and with elevation (new analysis added at L357-359), and found that sediment carbon did not change over time or with depth (thus not supporting there being big changes in carbon content as the site developed) but had slight variation with prior land-use. We justify treating new sediment as a single population when scaling up because of the lack of change over time or with depth in L245-251 of the methods, where we have improved to the text to make this clearer, and discuss the implications of this in the discussion (L442-464)*. *We acknowledge that there is some spatial variation in carbon content, but are wary of scaling up based on prior land-use as it is unclear whether that is having a causal affect (effects were weak, not consistent between TC and TOC, and the depth of new sediment means that it is unclear how past land-use could affect the new sediment).*

*The potential to refine estimates with knowledge of the spatial drivers of carbon stocks are discussed in L448-560. We lack the TOC data to assess changes over time (acknowledged in L363), but have added analysis of how this varies spatially (L359-361).*

5). Figure 5 includes a line which needs some more explanation. Line 401 states it represents an estimate of the construction carbon costs, without an explanation given how the cost is calculation and fitted in with the x- and y-axes of the graph. The legend to the figure states the line shows the carbon accumulation rate needed to break even with cost, which is something different and also not clear how it was derived.

*RESPONSE: Figure 5 (now Figure 7) solves the total carbon accumulation for different combinations of sediment carbon density and sedimentation. So each pixel on the graph shows the carbon accumulation for that combination of sediment carbon density and sedimentation. The line on the graph joined combinations of sedimentation and sediment carbon content where carbon accumulation (per ha per year) is equal to the construction carbon costs at Steart (lines 377-388, converted to a per ha value by dividing by site area). However, given the concerns raised by reviewers about other uncertainties in the carbon budget we feel this calculation of break-even time may be too simplistic and have therefore removed the line.*

6). Table 2 is rather wordy and would be more useful if more succinct, so that it can be more useful as guidance for other management realignment projects.

*RESPONSE: The “rationale and its implications” column is detailed as it provides an assessment for each uncertainty of its likely effect on our results. Given R2’s concerns about including necessary caveats we believe this detail should remain. However, we have shortened the “element” column so that provides a succinct description of the different uncertainties to help guide other projects.*

Minor edits:
Line 30: reword ‘…higher although more similar…’

*RESPONSE: Changed to “somewhat higher than”.*

Line 39/40: the first sentence of the Introduction is not very clear

*RESPONSE: Changed to “Earth’s ecosystems take up more carbon from the atmosphere than they release into it, causing increases in atmospheric CO_2_ to be smaller than expected from fossil emissions and land-use change”*

Line 105 ODN = ?

*RESPONSE: Spelled out on first mention. This is Ordinance Datum Newlyn, which is the sea-level reference point from which UK elevations are measured.*

Lines451 and 453 spelling: allochthonous

*RESPONSE: Corrected.*

Reviewer #2:

This is certainly an interesting and important study assessing the potential for C credits through a managed realignment project focused on blue C. I do have the following main concerns:

1.) Considering the large uncertainties concerning the role of allochthonous OC and IC contributions, title and abstract of the study are misleading. The discussion on allochthonous and inorganic C should be elaborated on, and resulting caveats/implications should already be mentioned in the Abstract. Compare comments in the pdf file attached.

*RESPONSE: We agree that this is a major uncertainty from a carbon budget perspective. While there was already extensive discussion of this issue, we have changed the title to clearly reflect what we have been able to calculate (carbon accumulation, direct construction emissions), and have added a caveat about the origin of carbon to the abstract. We agree that these uncertainties mean that our calculation of payback time was too simplistic, so have removed these from the manuscript.*

2.) I don't see if and how organic C loss from the excavated material is being considered /factored into your calculations. Compare comments attached.

*RESPONSE: We agree that this is a potentially important additional factor. We were not able to quantify this, although we note that the Environment Agency carbon calculator does include an embodied carbon value for rammed earth (L386). We have added notes in the methods about the potential for carbon loss (and accumulation) following embankment construction (L295-305) and have added this uncertainty to Table 2.*

3.) More emphasis/discussion on the role of non-CO2 greenhouse-gases, particularly methane, should be included. Compare suggested citations added to the pdf attached.

*RESPONSE: We have added more discussion about the potential for methane emissions to change following restoration. See lines 489-502.*

4.) Methods: I advice to clearly demonstrate the quality of your elevation-change assessment based on Lidar data in the main text (instead of just referring to an Supplementary Figure). This data is crucial.

*RESPONSE: We have moved this analysis into the main manuscript, so the results section now starts with this comparison (L309-322, Fig. 3). In addition to this comparison, the quality of Lidar data has been compared against fixed control points by Serrato and Pontee (2019) (see L266), and the error from this was already propagated in our analyses (L261-266).*

5.) I am concerned about the inorganic C quantification. Please see comments attached.

*RESPONSE: Please see our response to the L177 comment (we have also explained in detail how our methods relate to those suggested by the Coastal Blue Carbon Manual in response to Ref1). The method we used is a well-established procedure and is designed to minimise the loss of labile OC. We used this over LOI as we have found the latter grossly overestimates OC due to structural water loss.*

6.) Figures/Result presentation: You are comparing C percentages. However, from a C crediting perspective it is more useful to compare C stocks = C densities (C mass / volume). You can have higher C percentage in one soil, but still a smaller or equal C stock (= density). Thus, I advice to change reporting and figures to C stocks or densities.

*RESPONSE: We appreciate there is some merit in presenting C density rather than C content and bulk density, but argue that there is also merit in separating these variables. Our separate treatment of these variables is partly pragmatic, as we have a larger sample of %C measurements than bulk density measurements, and it is better to present what we actually measured. We also think that it is interesting to present the controls on C density (%C and bulk density) separately, and have welcomed seeing these reported separately in other studies and been frustrated to read studies that only present C density. There is a further advantage that carbon content is a relatively invariant property of sediment, whereas carbon density will change over time as sediment swells and contracts in response to wetting and drying. Following the reviewer’s suggestion, we now follow-through the calculation of C density in the Properties of newly accreted sediment section of the results so this information is clearly available (L353-354).*

*In terms of scaling up to site-wide estimates, treating %C and bulk density separately in the error propagation analysis assumes that they are uncorrelated. We lack sufficient co-located samples to robustly test that from the Steart dataset, but can assess this using some unpublished data of ours from six managed realignment sites around the UK. In that dataset, %C and bulk density were uncorrelated (r = -0.007, P = 0.959). Assuming this also applies to Steart, this means our treatment of these variables as independent and orthogonal in the Monte Carlo error propagation is justified.*

Detailed:

L1 – rephrase far exceeds carbon in title

*RESPONSE: Title changed to “Rapid carbon accumulation at a saltmarsh restored by managed realignment exceeded carbon emitted in direct site construction”*

L 21 - This would then refer to allochthonous carbon, which is tricky from a C-crediting perspective (e.g. Needelman et al. 2018 Estuaries). Another imporant potential could arise from the suppression of methane emissions; compare studies from North America (Kroeger et al. 2017 Scientific Reports, Fargione et al. 2018 Science Advances). L33 - see above. This depends on whether allochthonous organic carbon counts or not. That is, the alterantive fate of allochthonous OC (i.e. if it had not accumulated in the marsh) needs to be clear. If the allochthonous OC would get sequestered anyway (independed of the restoration project, e.g. in the tidal flats or the deep sea) it should NOT count.

*RESPONSE: We have added the uncertainties about carbon origin and gas fluxes as caveats in the abstract.*

L37 (keywords) In the North American Literature, the term Natural Climate Solutions is commonly used (Griscom et al. 2017 PNAS).

*RESPONSE: Added.*

L43 - This clearly depends on the time scale considered. Your statement is true for soil C sequestration (i.e. long-term burial over millenia). Over shorter time scales, C capture in forests (e.g. in biomass) is greater or comparable.

*RESPONSE: The reference we cite here (McLeod et al. 2011) presents annual rates of C capture by intertidal ecosystems (Table 1 in that reference) and terrestrial forests (Table 2 in that reference) which show that on an annual basis coastal ecosystems uptake carbon much faster than terrestrial forests on a per unit area basis. These figures relate to the amount of C buried in soil each year. If we look at C capture in live biomass, tropical forests take in 2-3 t C per ha per year, while we find that Steart accumulates >19 t OC per ha per year, so this statement is still valid. We have changed our wording to “a faster rare per unit area” just to be clear that this comparison is on a per unit area basis.*

L66 - Please define. What is the difference? Stick with one of the terms if possible.

*RESPONSE: Strictly, sequestration is where carbon is added to a pool where it is stored long term, whereas we prefer to use carbon accumulation as that does not make assumptions about the long-term fate of carbon. However, on reviewing the introduction we note that we had not been consistent in our use of these terms and have therefore simplified our language to just refer to carbon accumulation here and to use consistent terminology elsewhere.*

L86 - The methods are quite well established. I would not over-emphasize the new combination here.

*RESPONSE: Our statement referred to the combination of approaches rather than an individual method (which we agree are all well-established). However, we have removed this statement.*

L87 - i dont think this adds much information concerning the type of lab methods. Please rephrase.

*RESPONSE: Changed to “laboratory analysis of sediment”*

L131 - Puccinellia and Aster would refelct low-marsh vegetation. Are there also natural high-marsh sites adjacent that could be used as reference? What are possible implications of only focusing on low-marsh environments? E.g. Succession toward high-marsh communities after years to decades after restoration and thus, changes in C burial rates.

*RESPONSE: Puccinellia and Aster both occur over a wide range of elevations in UK saltmarshes and are abundant around MHWS (see Fig. 1 in Sullivan et al. 2018 J Applied Ecology). This community therefore captures the mid and upper marsh, and the pioneer community captures the lower marsh.*

L177 - HCl treatment can lead to an underestimation of organic C / overestimation of IC. How can you be sure that this did not happen in your analysis? Did you run any samples on a total (OC/IC) analyzer to assess the quality of this assessment?

*RESPONSE: 1N HCl digests at 80C are a standard, peer-reviewed procedure, designed to minimise the loss of labile OC and therefore keep the OC / IC balance as true as possible. See Hilton et al., 2008 (DOI: 10.1029/2006GB002905), Galy et al., 2007 (DOI: 10.1111/j.1751-908X.2007.00864.x), France Lanord and Derry 1994 (DOI:* [*10.1016/0016-7037(94)90210-0*](https://doi.org/10.1016/0016-7037(94)90210-0)*) and Sparkes et al., 2015 (DOI:* [*10.1016/j.margeo.2015.02.013*](https://doi.org/10.1016/j.margeo.2015.02.013)*). In this particular study, OC loss via acidification was not quantified but is expected to be minimal. A sentence to acknowledge the fact that some acid-soluble organic carbon could be los, and that this produces conservative estimates of TOC, consistent with carbon accounting principles, has been added to the manuscript (L186-190).*

L217 - This is a SEB (sedimentation erosion bar) or SET (surface elevation table)? compare Nolte et al. 2013, Journal Coastal Conservation.

*RESPONSE: We used a sediment erosion bar method, modified to meet the requirements of site managers. Specifically, this involved one permanent stake and a removable bar and second supporting stake, which were established each time on a fixed compass bearing. We have now clarified this and made reference to Nolte et al.*

L220 **-** Please state here (or elsewhere in the main text), how well these assessments agree. This is crucial information.

*RESPONSE: We have moved the comparison of ground and Lidar measurements of elevation change into the main section of the manuscript (L314-322)*

L224 - It is unclear what C composition refers to. Please specify or rephrase.

*RESPONSE: This is the carbon content. We have rephrased to clarify.*

L226 - Percentage = C content (g C / g soil)? C density (g C / volume of soil) is the relevant parameter when comparing C stocks or calculationg C sequestration rates.

*RESPONSE: The carbon content and the bulk density of the soil are both important variables that collectively determine carbon density, and we think it is valuable to present each separately.*

L233 - Again, is this C content or density? This has important implications.

*RESPONSE: We have clarified that this refers to the carbon content.*

L240 - I advice to present a more thorough assessment of the accuracy of your elevation-change assessment. How does Lidar data compare with SET/EB data. Compare comment above.

*RESPONSE: We have moved the comparison from the SI into the results.*

L241 - Okay, now I see. this calculation for the accumulation rate is correct. However, irrespective of that, I advice to compare C densities ( = stocks), not contents, between treatments.

*RESPONSE: Please see our earlier response on this.*

L287 - This needs to be presented in the main manuscript.

*RESPONSE: This is now moved into the main manuscript.*

L310 - Density of content? This is critical when comparing stocks.

*RESPONSE: We now clarify that this is the carbon content.*

L333 - The fate of the C of the excavated material should be included in your calculation. Is there a change in soil organic matter decomposition rate in this material in response to excavation and deposition in a different (redox)environment?

*RESPONSE: We agree that this is a potentially important additional factor. We were not able to quantify this, although we note that the Environment Agency carbon calculator does include an embodied carbon value for rammed earth (L386). We have added notes in the methods about the potential for carbon loss (and accumulation) following embankment construction (L295-305) and have added this uncertainty to Table 2.*

L444 - Methane emissions could be dramatically reduced. Compare citations given in Abstract.

*RESPONSE: We have discussed this in more detail, including adding the suggested Fargione and Kroeger references. See lines 489-502.*

L451 - allochthonous. Check spelling. Useful citations indicting the important role of allochthonous OC contribution to SOC stocks in NW European minerogenic marhses are Mueller et al. 2019, (Ecosphere and MEPS) as well as van de Broek et al. 2018 Global Change Biol + L456 - studies by Mueller and Broek, see above, should be cited here

*RESPONSE: Spelling corrected. Mueller and van de Broek references have been added to the discussion.*

L458 - Do you have any data on stable isotopes or similar to assess the amount of allochthonous OC? I think you should put more emphasis on this point. In case most of your OC is allochthonous, and using a conservative C crediting approach, the restoration project would not have generated much of a C credit. This need a more prominent discussion AND should alreafy be mentioned caveat in your Abstract.

*RESPONSE: We have added this caveat to the abstract (LINES 34-36) and improved the discussion on this. We currently lack sufficient stable isotope data to robustly determine the origin of carbon at the site.*

L463 - Yours would be imported, right? What are the implications. Please elaborate on this point.

*RESPONSE: We have simplified the discussion, particularly to highlight the main uncertainties and so this text has been removed.*

L473 - A sentence like this is missing in your Abstract. Please state the uncertainties again.

*RESPONSE: We have added this caveat to the abstract. We have given the origin and alternative fate of carbon as an example of the uncertainties here.*
