# Supplementary material for: Rapid carbon accumulation at a saltmarsh restored by managed realignment exceeded carbon emitted in direct site construction
Source: PLoS One. 2022 Nov 30;17(11):e0259033. doi: 10.1371/journal.pone.0259033 (PMC9710768; doi:10.1371/journal.pone.0259033)
Supplement: Attachment — Submitted filename: Response to reviewers Revision2.docx [file pone.0259033.s004.docx]

# Mossman et al. Response to reviewers – revision 2

This paper presents a well-written analysis of sediment carbon dynamics associated with a managed realignment site. Of particular interest, the authors estimate the carbon emission value of site construction activities, a component of carbon budgeting that is rarely addressed in such studies. The combination of on the ground data collection and remote sensing-based approaches provides for a high spatial and temporal resolution analysis of change over time in sediment surface elevation. These data, in combination with carbon content analysis of sediment cores collected within the restored area are used to estimate the total carbon stock of the newly deposited sediments within the restored area. The authors do an admirable job of describing and justifying their methodology.

We thank the reviewer for their positive comments

The one criticism I have of this manuscript is that the interpretation leaves the reader wondering about how important the carbon burial in this system is. The discussion seems to oscillate between suggesting that the site could have a huge carbon benefit and that there’s really no way to tell how important this site is as a sink for carbon without an extensive amount of additional research – leading the reader to come to their own conclusions.

The authors note minimal revegetation of the site for much of the collection period and major importing of sediments. The evidence seems to indicate that the majority of carbon accumulated at this site is allochthonous which would be consistent with other managed realignment studies cited in this manuscript. I’d suggest that the allochthonous fraction will likely offset MOST of the carbon accumulation benefits of the site.

To claim that there is a benefit to importing and burying allochthonous carbon it is necessary to demonstrate that the carbon would have otherwise been oxidized. Unless that can be proven conclusively, the carbon burial at this site merely represents a lateral transfer of C from other nearby ecosystems, not a net benefit (and therefore not an offset to carbon emitted due to site construction). Certainly, over time, this site will produce net emission offsets as the vegetation grows in and begins burying carbon in place (assuming as noted, that methane production isn’t significant here). The sediment carbon profiles suggest that this is not yet occurring at this site.

We thank the reviewer for their insightful comments, with which we agree. We have substantially revised the discussion and conclusion to provide much more critical analysis of the source of the carbon at our site, and the likely implications. In particularly, we provide discussion of the likely allochthonous source of our carbon, and the issues with counting for this in carbon accounting.

The new discussion paragraph (Line 523-543) reads:

“A particularly challenging element of quantifying the full carbon budget of a restored saltmarsh, and one we have not quantified, is determining (a) the nature and origin of the carbon accumulated in sediment (autochthonous or allochthonous) and (b) the fate of that carbon relative to it’s fate in the absence of the restored site (preserved and sequestered in the long-term, oror decomposed and released back to the atmosphere). This is critical to establish the additionality test needed for carbon codes and offsetting credits (e.g. Verified Carbon Standard Methodology VM0033 [78]), where in simple terms, carbon credits should only be generated through the creation of new net sinks of atmospheric carbon. While long term preservation of autochthonous carbon can be counted as a carbon benefit, how to account for allochthonous carbon is more complex. Concerns regarding additionality have led to variable treatment of allochthonous carbon because creating a new apparent store could be depleting supply to an adjacent system (i.e. the carbon would have been stored elsewhere in the absence of the project). In mineraogenic salt marshes, the majority of organic carbon is allochthonous in nature [79]. Indeed, in the early evolution of the mineraogenic Steart Marshes covered by this study, given the rapid sedimentation rates and relatively low plant colonisation, the vast majority (if not all) of the OC is likely to be allochthonous. Whether this represents a new carbon store will depend on it’s nature and alternative fate in the absence of the project. For example, aged mineral-associated organic carbon derived from reworked soils or sediments would not represent a new carbon store [79, 80]. However, burial of organic carbon of recent biogenic origin (e.g. phytoplankton or plant debris) may represent a new carbon store if it results in higher preservation rates in comparison to remineralisation in the dynamic estuarine environment. Tools such as biomarkers [81] and stable isotopes are being developed to better.”

The revised conclusion now reads:

“Our results show that at Steart Marshes, fast rates of sediment accumulation and high sediment carbon content combine to result in exceptionally fast carbon accumulation rates. Carbon accumulation at Steart Marshes over the first four years following reinstatement of tidal flow is fifty times larger than the direct carbon costs of site construction. However, there are numerous uncertainties (e.g. origin and alternative fate of carbon, greenhouse gas fluxes) that would need to be resolved in order to move to a fully quantitative carbon budget for restored saltmarshes. Perhaps most importantly, most of this carbon accumulation is likely to be allochthonous, and may not therefore represent newly sequestered carbon. It is particularly urgent to determine the origin and fate of the organic carbon relative to it’s fate in the absence of the project.”

Ultimately, I think the data presented here, combined with similar results from other similar sites (the Bay of Fundy study reference here and Drexel et al 2020.) demonstrate clearly the significant role of allochthonous carbon at sites that are importing sediments. For accounting and crediting purposes, the most conservative approach would be to consider this the “background” carbon against which future additions can be measured. The difference between the C in sediment of the project area and that of the surrounding salt marsh (multiplied by the total sediment volume of the project area) could provide a reasonable estimate of the likely carbon burial value of this site if one assumes that given enough time, it will reach equivalence with the natural marsh.

There is valuable data in the manuscript which will be of interest to PLOS readers but I feel like this contribution could be more impactful by more strongly emphasizing the allochthonous nature of the measured carbon and how what that means for its carbon value.

We thank the review for their positive comments on the manuscript. In addition to the revised discussion, we have firmed up our conclusion. The new conclusion now reads:

“Our results show that at Steart Marshes, fast rates of sediment accumulation and high sediment carbon content combine to result in exceptionally fast carbon accumulation rates. Carbon accumulation at Steart Marshes over the first four years following reinstatement of tidal flow is fifty times larger than the direct carbon costs of site construction. However, there are numerous uncertainties (e.g. origin and alternative fate of carbon, greenhouse gas fluxes) that would need to be resolved in order to move to a fully quantitative carbon budget for restored saltmarshes. Perhaps most importantly, most of this carbon accumulation is likely to be allochthonous, and may not therefore represent newly sequestered carbon. It is particularly urgent to determine the origin and fate of the organic carbon relative to it’s fate in the absence of the project.”

Note a few minor grammatical errors/misspellings:
82 “ including changes gas fluxes following”

Corrected to read “…including changes in gas fluxes…”

330 “eroision”

Corrected

331 “expecially”

Corrected

Reviewer #4: The authors provide what I consider to be a novel approach to look at C accumulation rates from a specific type of restoration – managed realignment, and compare these to the CO2 emitted from the actual effort required to restore the site. This type of information is needed to guide future restoration techniques to determine if the restoration is successful at returning C and to determine the environmental cost of that restoration. I think their approach is sound. At first I wasn’t sure why you didn’t measure the change in the silt layer above the previous land use in thee cores to calculate C accumulation, but then realized that the spatial representation is much better from the elevation models.

We thank the reviewer for their positive comments.

The only concern that I have is I would have preferred to have seen an intact site nearby if possible to act as a reference. It sounded to me like the natural reference site they used was one that was also a developing site, it’s described as a pioneer salt marsh that was bare with some Spartina anglica present. Using an intact reference site would have allowed them to determine if the initial pulse of sediment (0.449 m yr-1) in Table 1 was due to the low elevation of the restored site or an unusually high sediment load that occurred that year. Can you address this in the text as a caveat? Also, would it be possible to include the sedimentation rates from this natural site in Table 1?

We have added some extra information and analysis of the sedimentation change on the natural marsh. *In situ* measurements of that area of natural marsh to the south of the breach showed that this area did accrete but much more slowly than on the restoration site (28 mm). Analysis of lidar has shown that between 2014 and 2018, an average of 28.1 mm of accretion had occurred over this area. However, an area of natural marsh to the north of the marsh, experienced no net change of this time (0.03 mm).

We have added the following additional text to the methods and results.

Line 219-221: “In addition, two polygons were created on natural marsh areas to the north and south of the breach (Fig. S2) and the elevation change between 2014 and 2018 LiDAR imagery was assessed.”

Line 334-337: “*In situ* measurements of elevation change on the natural marsh found an increase of 0.26 m in total between December 2014 and March 2017. LiDAR assessment of elevation change on the natural marsh south of the breach was 0.281 m and -0.003 m to the north of the breach between 2014 and 2018.”

Double check some of the numbers. The mileage for the restoration was 69,000 km? That’s a lot.

We agree that it is an extraordinary distance, but it is correct. It was calculated by our engineering co-authors, who designed the site, based on information from on-site contractors. 411,397m^3^ of material were transported to create the sea defences of the site. The Volvo A25D Articulated Dumper Trucks that were used to transport the material from the dug creek network to the sea walls could carry 10.7m^3^ of sediment (15m^3^ standard volume but including a bulking factor of 1.4 for a clay material, where bulking factor = volume after excavation/volume before). The distances from excavation sites (creek networks) to the dumping sites (points along the new sea defence) were modelled prior to site construction to ensure that there was sufficient material and these distances were used to quantify the total distance travelled. The bulk of the unsuitable material from the creek excavation was towards the northern end of the scheme. The design of the Steart scheme sought to minimise distances travelled and therefore accommodated more of the landscape fill in the northern end of scheme. We have included information on the fuel used in the construction (i.e. by the dumper trucks, excavators etc.) in Table S2, and have now added the information above to S2.

Also, based on the numbers you present, I estimated that the amount of carbon returned was only 50x greater than the amount of C used to restore the site, not 2 orders. But I could have missed something there.

Yes, apologies. We have amended the text to state it is 50x greater in the abstract and in the discussion.

Other than that, I only had a few suggestions below in the introduction. Overall, I thought it was a well written paper and I enjoyed reviewing it.

Rich MacKenzie

We thank Dr MacKenzie for their positive comments and helpful input


L43 Not all ecosystems contain substantial carbon stocks (see Alongi papers). I would suggest changing this to “can contain substantial carbon stocks”.

Revised as suggested.

L43 the way this is currently written, it reads as if carbon stocks currently store carbon out of the atmosphere. Plus, you say this already in the first sentence. I suggest changing to: They can also contain substantial carbon stocks largely derived from atmospheric carbon and these stocks are sensitive to changes in climate or land use.

Revised as suggested.

L46 sequestered is associated with fixing CO2 from the atmosphere. Consider “Both allochthonous and autochthonous carbon accumulate in saltmarshes, through the deposition of sediment and organic matter carried in by tides or through plant growth, respectively. Or something like that.

Revised as suggested. Text now reads:

Lines 46-48 “Carbon accumulates in salt marshes through both the deposition of	sediment and organic matter carried in by the tides (allochthonous carbon) and	through in-situ plant growth (autochthonous carbon).”

L49 you haven’t really gone into their importance aside from C. Consider Despite their large carbon stocks, ~50%...

Revised as suggested.

L51 degraded from what?

Lines 50-52. Edited text now reads “…degraded by pollution, invasive species and hydraulic alteration…” and we have added an additional reference to Gedan et al. 2009 Annu. Rev. Mar. Sci. 2009. 1:117–41

L57-59 Carbon as been recognized as a benefit of saltmarsh restoration. Wouldn’t this provide a further motivation for restoration instead of creation? Changing creation to restoration also ties this paragraph into the next one.

We have edited the paragraph to include references to restoration, as an alternative or as an addition to creation. The revised paragraph now reads:

Lines 53-61. “In response to losses of saltmarsh and its associated biodiversity, ‘no net loss’ policies have sought to protect remaining wetlands, restored degraded sites and create new habitat [11], contributing to over 100,000 ha of intertidal wetland restoration or creation over the last 30 years [12]. However, the pace of global wetland creation or restoration is not sufficient to offset losses, where a key barrier is the availability of project financing [13]. Payments for ecosystem services, such as flood protection or biodiversity, offer potential financial mechanisms for saltmarsh creation or restoration [14]. Carbon accumulation (and thus climate mitigation) has been recognised as a potential benefit of saltmarsh restoration, and could therefore provide a further motivation for site creation or restoration [15, 16].”

L60 accumulation in restored

Revised as suggested.

L60 delete Robustly. I think just any attempts to quantify the rate of carbon accumulation is badly needed!

Thanks for the comment – we agree! Revised as suggested.

L64-68 This is great and I completely agree. I also think comparing different restoration techniques at restoring C is needed. This is a big issue with mangroves. I would consider adding something like that here as well, that comparing different methods is needed, which is sort of the goal of this paper, to assess managed realignment.

Good point! We have now included content on the potential differences resulting from difference restoration techniques and the importance of studying this. The next now reads:

Lines 66-75. “While saltmarsh restoration could potentially compensate for loss of natural saltmarshes, given known differences in topography and ecology [20, 21], it may not be appropriate to assume that restored or created marshes will ultimately store carbon at a rate comparable to natural saltmarshes [22]. Furthermore, the methods used in site restoration will also likely affect the total carbon sequestration and/or the rates of accumulation. For example, in mangroves, naturally and artificially regenerated forests differ in their structure, tree diversity and regeneration rate [23], potentially leading to differences in carbon storage rates, and in saltmarshes sedimentation rates differ between sites restored by managed realignment compared to regulation of tidal inundation [24]. It is therefore important to determine any differences between carbon accumulation in natural and restored saltmarshes, and any differences between restoration techniques.”

L69-75 using radionuclides (210Pb) to date sediments is another method that is popularly used to quantify C accumulation (Drexler et al. 2019; Arias-Ortez et al. 2018; etc.)

Revised as suggested (Lines 81-83).

L82 changes in gas fluxes

Revised as suggested.

L81-87 – yes!! Great point that a lot of restoration projects fail to consider.

Thank you!

L142 what type of soil auger?

A gouge soil auger, Royal Eijkelkamp, The Netherlands, was used. Text revised to include this (Line 150).

L143 I am not familiar with the term surface silts. Consider changing to surface sediments?

Revised as suggested.

L149-150 how did you sample the sediment plate? I am also not familiar with the term sediment plate

Apologies for the confusion. Sediment plate isn’t a technical term – it was our attempt to describe the polygons of sediment that formed occasionally during very dry periods in summer. We have edited the text and have referred to Fig S1, where the phenomenon is visible, and have also edited the legend of Fig S1 to make note of this.

Revised text now reads:

“In dry conditions when extensive mud cracking had occurred (see Fig. S1 2015 image), coring resulted in significant sample loss. In these cases, the initial core was discarded, and we separately collected (and sectioned) the sediment polygon formed by the mud cracks, the depth of the mud crack was recorded, and deeper sediments were sampled by taking a core between mud cracks (with core section depth corrected to depth from surface).”

**“Figure S1.** Photographs of sampling areas (Sites A-D). Note sediment cracking in 2015 images when conditions were dry in June; cracks and the ‘polygons’ formed by the cracking were sampled (see methods)”

L256 can you please clarify that the bulk density was specific to the silt layer that resulted after the restoration?

Revised as suggested.

L380 wow! 69,573 km? Is that correct? That’s almost 7 round trips between London and New York City.

Please see response above.

L392 – on L373 you state that 34,672 tC has accumulated since the project, and in L388 that the estimated total construction emissions were 753 tC. Unless I missed something, two orders would 75,300. The amount of carbon that has accumulated is 50x.

Yes, apologies. We have corrected the mistake to say 50x

L395-400 can you explain possible mechanisms as to why you are seeing these differences? Are total suspended sediment greater near Steart Marshes than those other sites?

We do discuss the possible mechanisms in the next two paragraphs, and as there is lots to discuss, we are reluctant to merge this first paragraph with the others. To ensure that it is clear that we will subsequently be discussing the potential mechanisms for these difference, we have moved the following lines up to the end of the first paragraph. The paragraph now reads:

Lines 407-418: “We find that Steart Marshes managed realignment has rapidly accumulated carbon since the fronting flood defence embankment was breached, and that this carbon accumulation is 50 times greater than the estimated direct carbon costs incurred during site construction. The rate of carbon accumulation at Steart Marshes (TC = 36.6 t C.ha^-1^.yr^-1^, TOC = 19.4 t C.ha^-1^.yr^-1^) is considerably higher than has been found at other sites. In the Bay of Fundy, which like the Severn Estuary is hypertidal, carbon accumulation is lower but within the same order of magnitude at 13.29 t C ha^-1^ yr^-1^ [28], but rates at other sites are an order of magnitude lower than at Steart Marshes. For example, saltmarshes in eastern England were reported to accumulate carbon at a rate of 1.04 t C ha^-1^ yr^-1^ for the first 20 years following creation [25], while a recovering saltmarsh in Australia accumulates at a rate of 0.5 t C ha^-1^ yr^-1^ [47]. The rate of carbon accumulation in a restored saltmarsh is a product of the rate of sediment accumulation and the carbon density of that sediment, and we can look at both these elements to see if Steart Marshes is unusual compared to other restored sites.”

L496-502 – what were the salinities at your site? This would support or not support this statement that restoration can increase CH4 if salinities are high enough.

We do not currently have data on the salinities of the site, although we are in the process of collecting some. However, published data on estuarine salinity close to the site suggests that salinity of the tidal waters is >28 throughout the year.

We have added the following text:

Line 514-517: “Estuarine salinity close to our site is thought to be >28 ppt throughout the year [74], and these polyhaline conditions are reported to have lower methane emissions than in lower salinities [75].”
